# Is There a Role for Treating Inflammation in Moyamoya Disease?: A Review of Histopathology, Genetics, and Signaling Cascades

**DOI:** 10.3389/fneur.2013.00105

**Published:** 2013-08-14

**Authors:** Adam M. H. Young, Surya K. Karri, Christopher S. Ogilvy, Ninghui Zhao

**Affiliations:** ^1^Department of Neurosurgery, Harvard Medical School, Massachusetts General Hospital, Boston, MA, USA; ^2^School of Clinical Medicine, Addenbrooke’s Hospital, University of Cambridge, Cambridge, UK

**Keywords:** moyamoya, brain, inflammation, genetics

## Abstract

Moyamoya disease is a slowly progressing steno-occlusive condition affecting the cerebrovasculature. Affecting the terminal internal carotid arteries (ICA) and there branches, bilaterally, a resulting in a fine vascular network in the base of the brain to allow for compensation of the stenosed vessels. While there is obvious evidence of the involvement of inflammatory proteins in the condition, this has historically not been acknowledged as a causal factor. Here we describe the fundamental histopathology, genetics, and signaling cascades involved in moyamoya and debate whether these factors can be linked as causal factor for the condition or whether they are simply a secondary result of the ischemia described in the condition. A particular focus has been placed on the multitude of signaling cascades linked to the condition as these are viewed as having the greatest therapeutic potential. As such we hope to draw some novel insight into potential diagnostic and therapeutic inflammatory targets in the condition.

## Introduction

Moyamoya disease is a slowly progressing steno-occlusive condition affecting the cerebrovasculature. Affecting the terminal internal carotid arteries (ICA) and there branches, bilaterally, a resulting in a fine vascular network in the base of the brain to allow for compensation of the stenosed vessels (1). The most distinguishing feature of this condition is the progressive stenosis of the ICA which induced further dilation of the perforating arteries that function as collateral pathways. Moyamoya disease is rare, with the incidence highest in East Asia of 0.35–0.94 and a prevalence of 3.16–10.5 per 100,000 (2, 3). The annual incidence is reported to be approximately 10% that of Japan (4). A bimodal age distribution has been observed with the predominant peak originally occurring at 5 years of age followed by a lower peak at 40 years (3).

There is significant debate over the pathogenesis involved in moyamoya disease, while there is obvious evidence of the involvement of inflammatory proteins, this has historically not been acknowledged as a causal factor in the condition (5). Here we describe the histopathology, genetics, and signaling cascades involved in moyamoya and debate whether these factors can be linked as causal factor for the condition or whether they are simply a result of the ischemia described in the condition. As such we hope to draw some novel insight into potential diagnostic and therapeutic inflammatory targets in the condition.

## Histopathology

The histopathology of moyamoya disease has stirred interest for a number of years. Stenotic changes occur in the intracranial ICAs immediately distal to the bifurcation. Progression later involves the proximal anterior and middle cerebral arteries and on rare occasions the posterior circulation may become involved. In adults, Weinberg et al. (6) describe a typical pattern of fibrocellular thickening of the tunica intima with excessive proliferation of the vascular smooth-muscle cells, marked tortuousness of the internal elastic lamina and attenuation of the tunica media (7). Moyamoya vessels have fibrin deposits in their walls, fragmented elastic laminae, attenuated media, and microaneurysms (8). Thrombosis as a result of collapse of the lumen is frequently observed in the vessels of patients (9).

This particular pathology is considered to be responsible for the onset of both ischemic and hemorrhagic stroke in these patients. Interestingly, the consensus view is that at a histological level these vessels lack inflammatory change, which has been considered to rule out an inflammatory component to the condition (5). However, Masuda et al. (10) noted the infiltration of macrophages and T cells in non-stenosed areas of the vessels, suggesting that the microthrombi may be a result of the chronic inflammation rather than a cause. In either case, the observation of microthrombi is not specific to moyamoya disease and therefore is unlikely to provide a complete explanation for its pathogenesis.

Although limited by the number of cases involved, there appears to be consensual evidence of inflammation in moyamoya. The lack of animal models makes it difficult to ascertain whether these findings contribute to the induction of the condition. Nevertheless, with growing evidence that inflammation is present in the vessel walls the debate of whether this process induces or results from moyamoya may be somewhat academic. The important factor is that there is reversible process in the vessels contributing to stenosis and as such significant questions have to be raise about whether anti-inflammatory agents could play a role in the treatment of the condition.

## Genetics

Moyamoya disease has a high familial occurrence accounting for up to 15% of affected patients (11). The female to male ratio in familial moyamoya disease is 5.0, which is much higher than that in sporadic cases (1.6). The mean age at onset of familial moyamoya disease (11.8 years) is significantly lower than that in sporadic cases (30.0 years). Interestingly, among parent–offspring pairs, the age at onset of offspring is on average 23 years lower than of parents, suggesting strong association with anticipation in familial moyamoya disease (12).

A number of linkage analysis have demonstrate the involvement of inflammatory genes in moyamoya disease. In particular, Ikeda et al. (13) demonstrated an association with chromosome 3 and specifically the locus responsible for the maintenance of vascular wall homeostasis. Chromosome 3p a principle site of proteins involved in multiple signaling cascades most notably the *IL5RA* (interleukin-5 receptor alpha), *TGFBR2* (transforming growth factor beta receptor II), *THRB* (thyroid hormone receptor beta), *RARB* (retinoic acid receptor beta), and *PPARG* (peroxisome proliferator-activated receptor gamma) are all involved in intricate signaling pathways which control and regulate angiogenic and inflammatory pathways.

Similarly, an association with human leukocyte antigens (HLA) (14) located on chromosome 6, the 6q25 marker was shared by 84% of families in a recent study. HLA has a strong connection with immune disorders (15). In particular, alterations in gene transcription and protein folding have been linked to aberrant expression of endothelial-leukocyte adhesion molecule-1 (E-selectin or ELAM-1), vascular cell adhesion molecule-1 (VCAM-1), and intercellular adhesion molecule-1 (ICAM-1) is induced by the inflammatory cytokines interleukin-1 beta (IL-1β) and tumor necrosis factor-alpha (TNF-α) via the activation of the pro-inflammatory transcription factor nuclear factor kappa b (NF-κB) (16).

It is clear that there is a panopoly of genes activated in moyamoya disease which have an inflammatory association. Whether, these are responsible for induction of the condition or a result upstream change remains unclear. Very little in moyamoya disease has been translated into animal models. Most recently, the discovery of RNF213 as a susceptibility gene has stirred some interest (17, 18). RNF213 encodes a gene finger protein with an AAA ATPase domain and is abundantly expressed in spleen and leukocytes (17). An RNA *in situ* hybridization analysis of mouse tissues indicated that mature lymphocytes express higher levels of Rnf213 mRNA than their immature counterparts (17). Recent studies have suggested that the postnatal vasculature can form through vasculogenesis, a process by which endothelial progenitor cell are recruited from the splenic pool and differentiate into mature endothelial cells (19). Levels of endothelial progenitor cells in the peripheral blood are increased in moyamoya disease patients (20). It is postulated that RNF213 may be expressed in splenic endothelial progenitor cells and mutant RNF213 might dysregulate the function of the endothelial progenitor cells (17). However, further research is necessary to elucidate the role of RNF213 in the etiology of the condition.

## Signaling Cascades

Cellular signaling cascades provide the interface of genetic and environmental interaction. An understanding of the cellular signaling cascades which are involved in a condition provides a platform for the identifying both diagnostic and therapeutic targets. This stems from the understanding that infection may play a key role in the pathogenesis of moyamoya disease (21).

The aberrant expression of mitogens, adhesion molecules, and angiogenic factors (22–25) and/or alterations in cellular responses to growth factors and cytokines demonstrate the involvement of inflammatory and hematopoietic cascades in vascular cells (26). This has been postulated to play a crucial role in the development of moyamoya pathogenesis (10).

### Vascular endothelial growth factor

Vascular endothelial growth factor is a 45-kD homodimeric, basic glycoprotein that requires association with heparin in order to function (27). It plays a central role in pathological vasculogenesis and vascular permeability in intracranial lesions. Similarly, VEGF has been shown to promote angiogenesis in the setting of cerebral ischemia (28). Interestingly, VEGF expression has been observed to being NF-kappa B dependent in a number of tissues, including endothelial cells (29). Furthermore, studies have shown the upregulation of Prox1 (30) and downregulation of Notch-1 (31) to have correlating effects on angiogenic processes. This regulation of angiogenesis by a pro-inflammatory transcription factor has drawn insight into the potential signaling cascades available for manipulation in moyamoya disease.

Aberrant expression of VEGF is evident around the affect vasculature in moyamoya disease. In a small study, Sakamoto et al. (28) observed a fourfold increase in VEGF expression in patients with moyamoya disease. More specifically, the VEGF −634G allele has been identified has having a particularly strong influence on moyamoya disease and poor collateral vessel formation (32). The expression of VEGF is not limited to the cerebral vasculature in moyamoya disease, Takekawa et al. (33), describes the growth factor in glial cells and Sakamoto et al. (28) in the dura matter. The authors suggest that this displays evidence of the pathological mechanisms extending beyond the cerebral vasculature. Nevertheless, this is most likely associated with the induction of pro-inflammatory cascades as a result of ischemia and a secondary marker of disease rather than a primary modality for the pathogenesis.

### Basic fibroblast growth factor

Basic fibroblast growth factor (bFGF) is an 18-kD protein consisting of 146 amino acids (34). The primary role of bFGF involves the stimulation of mesodermal and neuroectodermal proliferation (35), additionally it has been shown to induce growth of vascular smooth muscle and, when combined with VEGF, can play a leading role in angiogenesis (36). A hypothesized pathway for this is via the upregulation of circulating chemokines (37). In particular, the chemokine-mediated regulation of angiogenesis is highly sophisticated and fine tuned, and involves pro-angiogenic chemokines, for instance, CXCL8/IL8 interacting with the CXCR2 receptor, and anti-angiogenic (i.e., angiostatic) chemokines, for instance, CXCL10/IP10 interacting with the CXCR3 receptor (37).

Basic fibroblast growth factor has been observed to be aberrantly expressed in the colony-stimulating factor (CSF) of moyamoya disease patients (24, 38), with both groups agreeing that the expression levels were 10-fold higher in moyamoya disease. Additionally, bFGF was also observed in the thickened tunica media, assisting suggestions that the upregulation of the molecule is associated with both stenotic and angiogenic processes (38). Yan et al. (39) describes separate *in vitro* and *in vivo* models of bFGF promoting neovascularization. In relation to corneal wound healing, a comparison with recombinant human epidermal growth factor (rhEGF) led the others to believe that the effects of bFGF were too strong to promote controlled healing (39). Interestingly, the inhibition of bFGF has been demonstrated to inhibit the proliferation and migration of endothelial cells (40). As such it could be viewed that bFGF plays and intricate role in the development of vessel proliferation and endothelial cell recruitment. With this in mind there may be future scope to incorporate the molecule into new therapeutic strategies.

### Hepatocyte growth factor

Hepatocyte growth factor (HGF) is one of the largest disulfide-linked cytokines, and in humans the protein is synthesized as a single-stranded 728 amino acid protein (6). The proteolytic activation of HGF involves the release of a 31 amino acid N-terminal signal peptide which has been observed to potentiate the growth of various epithelial, endothelial, and mesenchymal cells (41).

In various injury and disease models, the HGF-Met pathway plays a critical role in acute tissue protection and regeneration, and in providing less susceptibility to chronic inflammation and fibrosis (42).

Nanba et al. (43) demonstrated a twofold increase in both HGF and its receptor c-Met expression in the tunica media and intima of patients with moyamoya disease compared to control groups with cervical spondylosis and unilateral internal carotid artery occlusion. From this it was postulated that the upregulation of HGF plays a role in the pathogenesis of intimal thickening and vascular smooth-muscle cell migration. Additionally, hypoxia inducible factor-1α, which promotes smooth-muscle cell proliferation in the presence of bFGF and HGF, is present in elevated levels in moyamoya disease (44). In addition to HGF being densely found in the carotid fork, its CSF level is markedly elevated in moyamoya disease, suggesting that HGF may be a key protein for pathogenesis of moyamoya disease (43). From this information it is apparent that the inhibition of HGF in the carotid vasculature would be advantageous. Whether it plays a role in the initiation of other cascades remains uncertain however, it is clear that inhibiting HGF expression in the carotids could prove beneficial in the treatment of moyamoya disease.

### Transforming growth factor-β1

Transforming growth factor-β1 (TGFβ1) in its original form is a 390 amino acid peptide that is proteolytically activated to form the active 112 amino acid monomeric form of the active TGFβ1 homodimer (6). Implicated in a variety of cellular processes including cell growth, proliferation, and differentiation (45), TGFβ1 is involved in the expression cascade of various connective-tissue genes at normal physiological concentrations. Nevertheless, when it is aberrantly upregulated it has been postulated to contribute to pathological angiogenesis (46).

The upregulation of TGFβ1 has been implicated in the pathogenesis of moyamoya disease. Specifically, Hojo et al. (46) demonstrated a threefold increase in serum TGFβ1 levels of moyamoya disease patients compared to controls. Additionally, similar studies on atherosclerosis failed to demonstrate significant deviation from control results (47), speculating that TGFβ1 may play a significant role in the neovascularization process (46). Furthermore, TGFβ1 has been associated with increased production of elastin synthase, which is involved in intimal cell proliferation, a hallmark of moyamoya disease (25). Interestingly, a recent study by Liu et al. (48) failed to observe any aberrant gene expression when sequencing the first exon of TGFβ1 in both European and Japanese cohorts. In particular, they failed to demonstrate the previous association of rs1800471 and tendency toward significance of rs1800470 suggesting that although TGFβ1 may be aberrantly expressed at the protein level this may be a result of stimulation from upstream mediators rather than mutations within the gene itself (48).

### Granulocyte colony-stimulating factor

Granulocyte colony-stimulating factor (G-CSF), key mediator of the acute inflammatory reaction exists as a 174 amino acid mature protein weighing 19.6 kD (49). It is a glycoprotein, growth factor, and cytokine, the main function of which is the stimulation of proliferation, survival, and maturation of neutrophil precursors and mature neutrophils. The regulation of such properties by G-CSF is via the activation of Janus kinase (JAK)/signal transducer and activator of transcription (STAT) and Ras/mitogen-activated protein kinase (MAPK) and phosphatidylinositol 3-kinase (PI3K)/protein kinase B (Akt) signal transduction pathways (50). The activation of NF-kappa B is also heavily linked to the regulation of the cytokine (51).

In moyamoya disease elevated concentrations of G-CSF have been demonstrated to be as high as 1.5-fold more than controls (52). Interestingly, expression of CSF was also observed within atherosclerotic plaques (53). As such it is unlikely to be specific to moyamoya disease however, would appear to contribute to vessel narrowing because of its action on cell recruitment (53). However, the extent to which G-CSF is involved in the pathogenesis of moyamoya disease has yet to be determined.

### Extracellular markers

Individually, prostaglandin E2 (25), IL-1β (25), and cellular retinoic acid binding protein 1 (54) have been shown to be increased in concentration. Additionally, several soluble endothelial adhesion molecules have been observed in the CSF of patients with moyamoya disease. Specifically, the observation of VCAM-1, ICAM-1, and E-selectin (23).

Additionally, levels of MMP-9 were found to be significantly raised in moyamoya disease comparison to healthy controls (55). MMP-9 (gelatinase B) attenuates the impenetrability of the blood-brain barrier by interrupting the endothelial basal lamina and as a consequence plays a role in cerebral ischemia, and the formation and rupture of cerebral aneurysms, as well as other CNS pathologies.

## Conclusion

There is significant debate over the pathogenesis involved in moyamoya disease. While there is obvious evidence of the involvement of inflammatory proteins, this has historically not been acknowledged as a causal factor in the condition (5). Here we have reviewed the histopathology, genetics, and signaling cascades involved in moyamoya disease identifying a number of key targets which may assist in the treatment of the condition. Although it remains uncertain to whether these factors play a role in the initiation of signaling cascades or if they are downstream mediators the clearly play significant roles in the pathogenesis. With this in mind it is important to consider these as important targets in the treatment of moyamoya disease.

In particular, significant research will have to be undertaken to fully understand the effects of each signaling molecule and at which part of the pathway they act. The distinct creation of moyamoya vessels is almost certainly secondary to the initial stenosis observed in the ICA. By preventing this it is possible that the subsequent creation of fragile vessels could be avoided. From this review it is apparent that bFGF and G-CSF play a role in this process and could both demonstrate potential diagnostic and therapeutic relevance in the future.

## Conflict of Interest Statement

The authors declare that the research was conducted in the absence of any commercial or financial relationships that could be construed as a potential conflict of interest.
